# Myths and methodologies: Invasive and non‐invasive assessment of respiratory muscle activity in humans

**DOI:** 10.1113/EP091526

**Published:** 2025-03-27

**Authors:** Anna L. Hudson, Caroline J. Jolley, Simon C. Gandevia, Jane E. Butler

**Affiliations:** ^1^ Flinders Health and Medical Research Institute Flinders University Bedford Park South Australia Australia; ^2^ Neuroscience Research Australia Randwick New South Wales Australia; ^3^ The University of New South Wales Sydney New South Wales Australia; ^4^ Centre for Human & Applied Physiological Sciences, School of Basic & Medical Biosciences, Faculty of Life Sciences & Medicine King's College London London UK; ^5^ Department of Respiratory Medicine King's College Hospital NHS Foundation Trust London UK

**Keywords:** diaphragm, electromyography, intercostal muscle, single motor unit

## Abstract

Survival relies on neural respiratory drive from the medullary respiratory centres to activate the respiratory muscles for breathing. Accurate assessment of respiratory muscle activity, as an estimate of neural respiratory drive in humans, will facilitate a greater understanding of respiratory physiology, the pathophysiology of diseases and injuries, clinical investigations and the neurophysiology of breathlessness perception. Here, we highlight the methodologies to measure respiratory muscle activity, their appropriate application, the potential limitations of the data and their safety and feasibility considerations. These recommendations can be applied to all skeletal muscles.

## Abstract (highlights)

- **What is the topic of this review?**
  We describe the invasive and non‐invasive methods to measure human respiratory muscle activity, based on current evidence and experience. We caution against the ‘myth’ that respiratory muscle activity equals neural respiratory drive but acknowledge that selection of methodology is a balance between the best estimate of neural respiratory drive together with safety and feasibility.
- **What advances does it highlight?**
  This review highlights the most appropriate inspiratory muscles and method to estimate neural respiratory drive. It also reveals how recent methodological innovations (multi‐channel/high‐density EMG recordings) might enable more accurate estimates of neural respiratory drive (i.e. single motor units), but in a non‐invasive way.

## 1 INTRODUCTION

Respiratory diseases are common, constituting ∼16% of the total disease burden worldwide in 2021, and are unlikely to decline given our ageing population, climate change, increased risk of global pandemics, a new generation of nicotine addiction (electronic cigarettes/vapes), spinal cord injuries and more (Institute for Health Metrics & Evaluation, 2024). We measure respiratory muscle activity to estimate neural respiratory drive in health (physiology) and in disease or injury (pathophysiology), to assess the pathophysiology of neuromuscular disorders (neuropathy and conduction tests) and to measure respiratory reflexes, for example. How can we assess respiratory muscle activity accurately, safely and in a practical way for clinical use?

**FIGURE 1:**
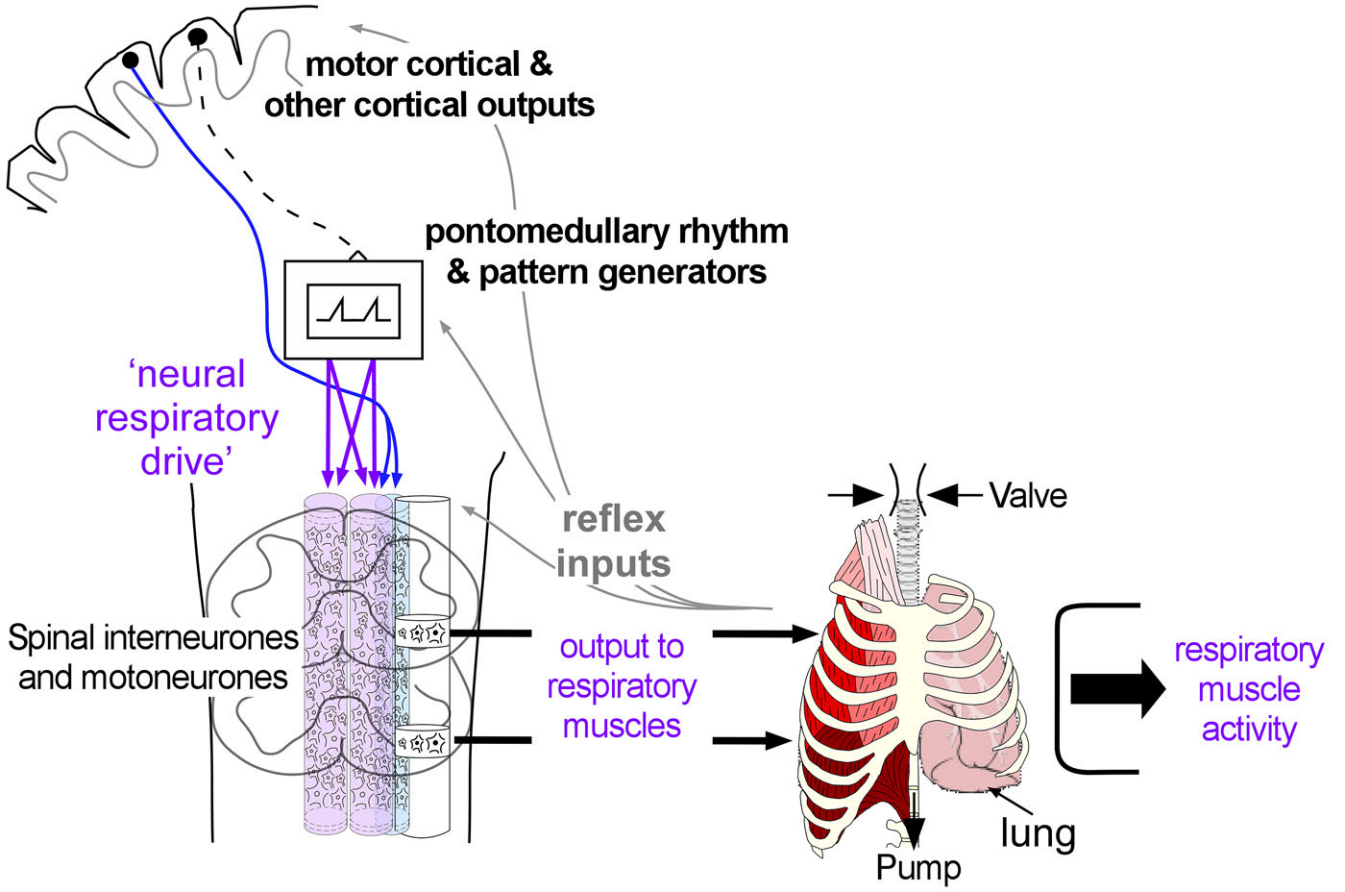
Neural control of respiratory muscles. Respiratory motoneurones in the spinal cord receive synaptic input from rhythmic respiratory centres in the pontomedullary region for breathing, but also from other sources, such as descending corticospinal tracts and reflex inputs. These can reach the motoneurones directly or via spinal interneurones, which are likely to be important to coordinate the activation across synergist ‘valve’ muscles that maintain airway patency and synergist ‘pump’ muscles that generate negative intrathoracic pressures (see Hudson et al., 2019). The final common pathway is the motoneurone, and by recording motoneurone output with different EMG techniques, we can estimate neural respiratory drive. In comparison to skeletal muscles in the limbs, when selecting which EMG technique to use we also need to consider the unique anatomy of the respiratory muscles and underlying lung.

The assessment and interpretation of the different methods to measure the EMG from respiratory muscles require the same considerations as for EMG from the skeletal muscles in the rest of the body. A recent series of papers provides general background and recommendations (see Besomi et al., 2019 and others from the ‘CEDE’ project), but the assessment of respiratory muscle activity brings additional challenges, relating, in part, to their critical function of breathing. There are at least three additional factors to consider in the assessment of respiratory muscle activity. The first factor is their anatomy. Respiratory muscles wrap around the three‐dimensional rib cage, with directly adjacent agonist and antagonist muscles (e.g. the external and internal intercostal muscles, cf. the biceps and triceps muscles on opposite sides of the humerus) and/or are deep, hence more difficult to access (e.g. crural diaphragm; Figure 1). In addition, their position close to the lung brings safety considerations, and their position close to the heart brings technical difficulties because respiratory EMG methods also record the ECG. The second factor is their rhythmic activity. Respiratory muscles have a high duty cycle and in health are not electrically silent for more than ∼1–2 s, if at all (see Saboisky et al., 2007), during resting breathing. Even during voluntary breath holds, involuntary phasic diaphragmatic contraction starts after ∼30 s (depending on arterial partial pressure of CO_2_) (Agostoni, 1963). The third factor is their multifunctionality. In addition to rhythmic activation for breathing, respiratory muscles contract for speech, protective reflexes, such as coughing, sneezing and swallowing, and non‐respiratory movements, such as trunk rotation (Figure 1). Reflexes mediated by chemo‐ and proprioceptors also act at all levels of their neural control. All these potential inputs can complicate the measurement of respiratory muscle activity and interpretation of the output of respiratory motoneurones as neural respiratory drive.

This review focuses on the assessment of inspiratory pump muscle activity because this is the key component of respiratory control to overcome the recoil pressures of the lung and chest wall to inflate the lungs. Upper airway and expiratory muscles are considered where relevant. Although theoretically, the same assessment methodologies can be used in the paediatric population, our recommendations stem from our experience in adult populations. We have not summarized methodologies to record evoked responses from respiratory muscles but refer readers to previous major reviews (e.g. American Thoracic Society/European Respiratory Society, 2002; Laveneziana et al., 2019).

## 2 FIRST PRINCIPLES

**FIGURE 2:**
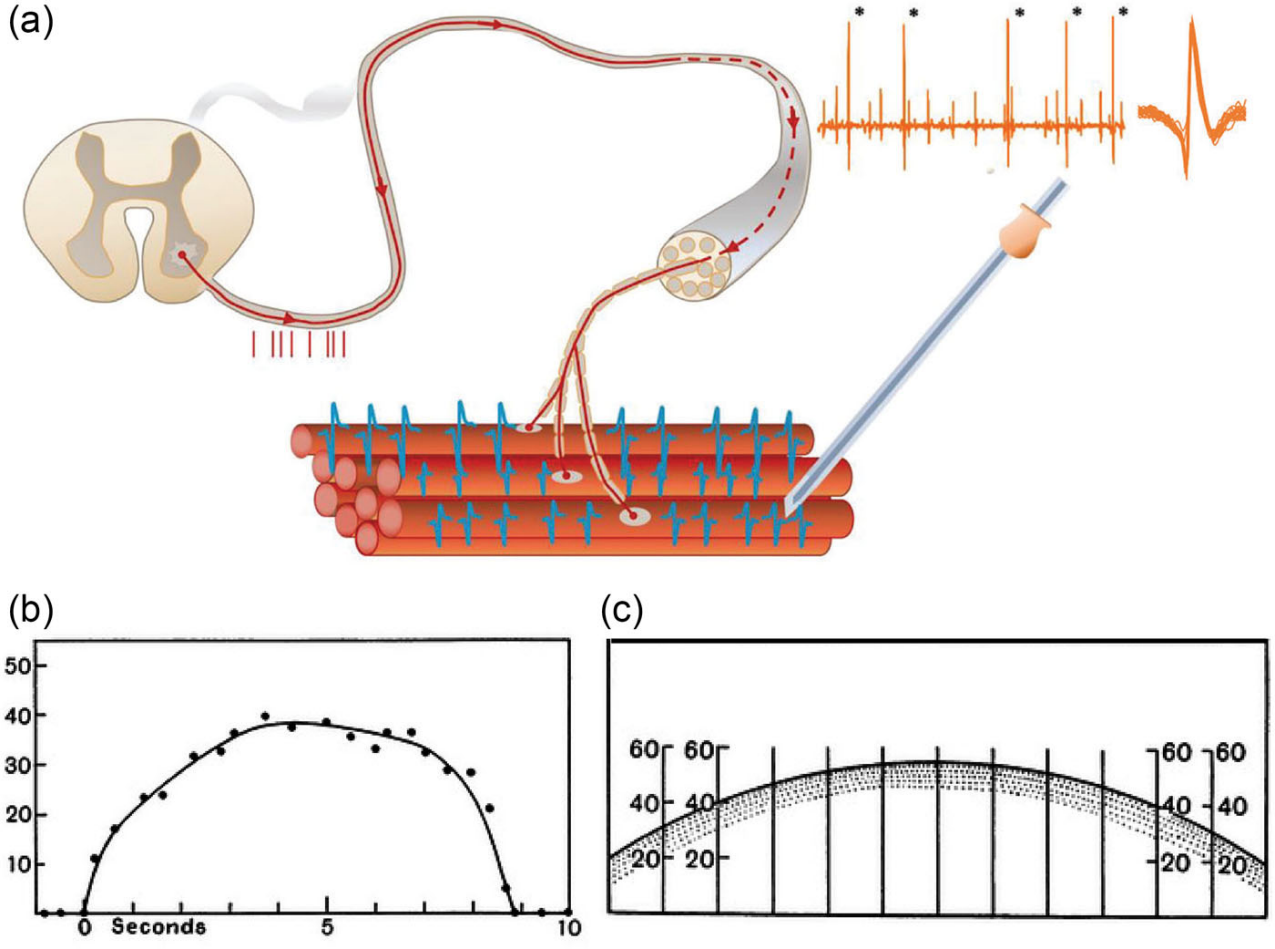
First principles: the motor unit and the recruitment and rate coding of motor units for force production. The one‐to‐one relationship between discharge of an α‐motoneurone in the spinal cord and action potential in muscle fibres in the periphery has been recognized and recorded in humans for ∼100 years. (a) Single motor unit recordings using a needle electrode. Action potentials recorded in the muscle fibres reflect the impulses in the nerve following motoneuron depolarization above threshold. (b) Typical behaviour of a single motor unit upon activation, that is, recruitment of the motor unit (discharge rate exceeds 0 Hz, as shown on the *y*‐axis) and increased discharge (rate coding) with time (shown on the *x*‐axis). The discharge of a motor unit makes muscle tension (also known as twitch force), and force increases with increases in neural drive to a motoneuron pool via recruitment of more motor units and higher discharge frequency of already active motor units (rate coding), which results in force summation. (c) Activation of a motoneurone pool. Each vertical line represents a motor unit, and the discharge rate of the unit is shown along the *y*‐axis of each line where the semi‐circle intersects the vertical line. The degree of excitation of the motoneurone pool is depicted by the semi‐circle. Many motor units are active, each with different discharge rates. Multiunit recordings (e.g. bipolar surface EMG) capture the activity of multiple motor units, and although they can indicate the approximate level of contraction, these recordings contain less precise information. Figure adapted from Farina and Gandevia (2024).

As skeletal muscles, respiratory muscles comprise a collection of motor units, with muscle fibres that are intermingled with overlapping territories and innervated by an α‐motoneurone in the spinal cord. When the motoneurone in the spinal cord is depolarized above threshold potential, an action potential propagates down the axon and activates the muscle fibres (see Figure 2). The mechanical properties of respiratory motor units range from smaller, slow‐twitch units to larger, fatigable fast units. The distribution of these fibre types can vary topographically and according to their mechanical properties (for more details, see review by Hudson et al., 2019). The principles remain the same for all muscles independent of the exact fibre‐type composition of the muscles. To make force, motor units are recruited (recruitment). To make more force, more motor units are recruited and/or the discharge rate of already activated units is increased (rate coding). How we interpret motor unit behaviour depends on what method is used to record it. Motor unit territories in the respiratory muscles have not been studied thoroughly. In cats, the territories of a single diaphragm motor unit are estimated to span 10%–15% of the total costal–sternal diaphragm area (Fournier & Sieck, 1987). Again in cats, the territories of diaphragm units were reported to be smaller than those in the hindlimb, but it was not determined whether this was attributable to lower innervation ratios (that might reflect smaller motor unit territories) or for other reasons, such as smaller cross‐sectional area or lower specific tension of diaphragm fibres (Sieck & Fournier, 1987). Early estimates of human motor unit territory size of 5–10 mm in the biceps and tibialis anterior muscles (Stålberg, 1979; Stalberg & Antoni, 1980) are consistent with more recent data from the genioglossus muscle (a ‘valve’ muscle innervated by the hypoglossal motor nucleus) using new EMG recording technology (Luu et al., 2018).

## 3 THE ‘MYTHS’

It is a myth that respiratory muscle activity equals neural respiratory drive, because neural respiratory drive is the output from the premotor medullary centres and not respiratory muscle output or respiratory muscle activity (see Figure 1). Respiratory muscle activity is the best surrogate we have for recordings from humans, but the term ‘respiratory neural drive’ should be considered carefully because direct measures of the output of respiratory motoneurones are likely to be related to the neural respiratory drive that they receive, but it is not a direct measure of neural respiratory drive. However, to provide the best estimate of neural respiratory drive in humans, the closer the EMG measure is to the ‘source’, the better. Based on this concept, there is a hierarchy in EMG recording methodologies for respiratory muscles. Of the techniques currently used, the hierarchy is as follows: (i) intramuscular single motor unit recordings; (ii) intramuscular multiunit recordings; (iii) intra‐oesophageal catheter EMG recordings from the crural diaphragm; and (iv) surface EMG methodologies. This hierarchy is applicable to recordings from all skeletal muscles, excluding the intra‐oesophageal catheter, which takes advantage of the unique anatomy of respiratory muscles. However, the proviso of this hierarchy is safety and practical issues. For every study, selection of the appropriate methodology is a balance between the best estimate of neural respiratory drive together with safety and feasibility.

Previous methods that purported to measure/estimate respiratory neural drive have been shown to be inaccurate with hindsight and methodological advances. For example, that the amount of inspiratory EMG (intra‐oesophageal bipolar electrode‐mounted catheter) equalled neural respiratory drive was shown to be an error owing to muscle movement relative to electrodes (Gandevia & McKenzie, 1986) and that mechanical measures (from intrathoracic pressures) are good surrogates for respiratory muscle activity was shown to depend on the degree of neuromechanical coupling and thus does not hold true in states of impaired lung, chest or abdominal wall mechanics (Hudson & Laveneziana, 2015; Jolley et al., 2015). Of course, advancements in methodologies might reveal other errors in the future.

## 4 THE ‘METHODOLOGIES’ TO ASSESS RESPIRATORY MUSCLE ACTIVITY

### 4.1 Intramuscular techniques

Intramuscular recordings from respiratory muscles are performed with either a solid needle electrode or a pair of fine wire electrodes, hooked at their ends, that are inserted into the muscles using a needle (the needle is then withdrawn). Intramuscular recordings have been made from the costal diaphragm, the parasternal and the external intercostals, the scalenes and the sternocleidomastoid muscles. A monopolar needle (recording area 0.34 mm^2^) is most commonly used with a reference surface electrode placed nearby on the skin (Gandevia et al., 2006; Hudson et al., 2010, 2017; Nguyen et al., 2019; Nguyen, Lewis et al., 2020). Depending on the recording area of the wires (i.e. how much is exposed by removal of the insulation), fine wires can be used for single motor unit (SMU) recordings (e.g. 0–1 mm of insulation removed) or multiunit recordings (e.g. 2–3 mm of insulation removed). The principles for these SMU and multiunit fine wire recordings are comparable to those for solid needle electrodes for SMU measures (small, selective recording area; see Table 1) or those for bipolar surface EMG measures (larger recording area and multiunit recordings; see below), respectively. Intramuscular ‘SMU recordings’ are often but not always recordings of multiple motor units that can be discriminated into multiple SMUs off‐line, if guidelines for ‘proper use’ are followed (see Table 1). It is unlikely that all fibres of a motor unit are recorded by the needle or wires; instead, a population of fibres that represents the motor unit is sampled. The number, their spatial arrangement and temporal activation give the SMU action potential its distinct shape and allow each one to be discriminated. From our experience, motor units from the intercostal muscles have much more distinct shapes than those from the diaphragm. This might relate to differences in motor unit territories in these muscles that might reflect anatomical or functional differences, but this remains speculation. It can be challenging to record SMUs from female participants if the rib spaces are narrower and muscles thinner. Hence, we have studied many more male than female participants (e.g. Gandevia et al., 2006; Hudson et al., 2010; Nguyen et al., 2019; Saboisky et al., 2007) and would have missed sex differences in respiratory motor control using intramuscular techniques (see Hudson et al., 2024).

**TABLE 1:** The technical principles underlying the assessment of respiratory muscle activity using intramuscular EMG.

Technical principles	Intramuscular EMG (needle and wires)
Details for proper use (calibration, validation, range, sensitivity, reproducibility, variability minimisation)	Calibration. For wires, standardize exposed recording area (impedance) across multiple recordings sites or normalize activity to EMG during resting breathing rather than maximal manoeuvres, which can be painful, dislodge electrode positioning or, in the case of solid needle electrodes, can be unsafe (e.g. Nguyen, Amirjani et al., 2020). Validation. The ECG is easily distinguishable from SMU activity, but more caution is required for multiunit recordings using wire electrodes. See also tips for effective use below. Range. An EMG amplifier with ability to change gain ‘on the fly’ for recording of SMUs is helpful because the size of the EMG can vary depending on needle proximity to the motor unit. Sensitivity. A high sampling rate is required for SMUs (≥10 kHz) and a lower sampling rate for multiunit recordings from wire electrodes (≥2 kHz). Reproducibility. SMU discharge properties (timing and rate) are comparable for one breath versus an average of three breaths in the diaphragm (ICC range 0.80–0.97) (Nguyen et al., 2019), for repeated protocols of three breaths for PSIC (ICC range 0.83–0.85) (Hudson et al., 2017) and repeated studies in PSIC muscles (Hudson et al., 2019: Fig. 2B). Variability minimization. Choose breaths with stable air flow and volume for analysis. If comparing activity in more than one respiratory muscle across different breaths (e.g. intercostal muscles in different interspaces), measure rib cage and abdominal excursions to check that breathing ‘strategy’ (ratio of rib cage to abdomen expansion) is comparable across recordings (e.g. Gandevia et al., 2006).
Tips for effective use	Image muscles and surrounding anatomy with ultrasound before and/or during electrode insertion and note both the lung excursion and the maximal muscle depth. For the diaphragm, electrodes can be inserted below the pleural reflection to reduce risk of pneumothorax (Amirjani et al., 2012). Online audio and visual feedback of EMG while searching for a site is recommended but not for the participant when recording. Optimize the quality of recordings online for successful discrimination of SMUs offline; for example, audible crisp discharges and large signal‐to‐noise ratio visually. Analyse only pain‐free recordings. To minimize any pain, inject a small volume (1–2 mL) of 1% lignocaine under the skin and in overlying muscles (not in the target muscle), target the widest gap between ribs and aim low in the interspace because the blood vessels and nerves run higher in the interspace. Record from multiple sites within the muscle (e.g. 10) for each participant to sample more motor units. The needle can remain subdermal, but the angle of the needle is changed with audio and visual feedback. Confirm the position of the needle/wires during resting breathing, but also using additional voluntary behaviours, such as asking the participant to push below usual end‐expiratory lung volume to check inspiratory versus neighbouring expiratory layers of muscle. For wires, ultrasound can be used while advancing the needle (Hodges & Gandevia, 2000), but given that the wires can move with needle removal, the recording site should be confirmed afterwards via EMG during inspiratory and expiratory behaviours. To verify the site, EMG can also be recorded by connecting the wires to an amplifier before removing the needle, if possible and safe.
How to avoid errors	Note the limitation of the SMU technique that quantifies neural drive by rate coding but does not fully capture motor unit recruitment, especially if comparing across respiratory muscles. Different inspiratory muscles are thought to have different strategies, for example, rate coding (diaphragm) versus recruitment (scalene and intercostal muscles) (Gandevia et al., 1999). It is possible to see recruitment of new motor units if the recording site is maintained (e.g. Hudson et al., 2010) or to use an estimate of recruitment of the average number of units per site (e.g. Gandevia et al., 2006). Take care when interpreting muscle activity because EMG from neighbouring muscles can be recorded (Demoule et al., 2003; Hodges & Gandevia, 2000). This is more problematic for multiunit recordings with wires than for SMUs with a needle. Needle electrodes can be unstable, and small movements can change motor unit morphology (e.g. size), which makes SMU identification difficult over long time periods. Any larger contractions should be isometric or quasi‐isometric to attempt to maintain the recording site and for safety (Hudson et al., 2010). Wires in the diaphragm can be secured and stable for ∼3 h of light activity/office work (unpublished observations). Wires are safer for higher‐force contractions because the wires are thin and flexible, and hooks help to maintain their position, in comparison to the solid needle electrode. Once the insertion needle is removed, wires can usually record only one site within the muscle. Confirm that the needle/wires are in situ at the end of each protocol and not in a different expiratory muscle layer by asking the participant to breathe out slightly below end‐expiratory lung volume.
Appropriate uses of techniques, with examples	SMUs from intramuscular electrodes are the definitive measure of respiratory motoneurone output and the best estimate of neural respiratory drive during resting breathing, with recordings from the costal diaphragm. These recordings allow investigations of precise neural control and strategies for motor unit recruitment (e.g. Hudson et al., 2017; Saboisky et al., 2007). These types of studies are more suited to healthy populations of participants, given the safety considerations. SMU recordings can be analysed to give motor unit action potential properties in neuromuscular disorders etc. This has been used to indicate motoneurone loss and motor unit remodelling in healthy ageing (Nguyen et al., 2019) and chronic tetraplegia (Nguyen, Lewis et al., 2020).
Inappropriate uses of techniques, with examples	Use without appropriate training, prior or simultaneous ultrasound imaging and/or following recommendations, which can lead to a pneumothorax (Podnar, 2013). Use in maximal contractions or larger breaths (with greater lung excursions), owing to pain and safety considerations, especially with a needle compared with wire electrodes. Ill‐considered use in people with reduced lung capacity and/or in certain protocols, for example, rotation is more risky than resting breathing. Recordings from costal diaphragm (especially wires with larger recording areas) can also record from internal intercostals activity in expiration, which might complicate interpretation (Hodges & Gandevia, 2000). If measuring multiunit activity, a large‐amplitude SMU (i.e. close to the needle tip) can exaggerate EMG amplitude estimates and should be excluded from analysis (unpublished observations; Nguyen, Amirjani et al., 2020).

Abbreviations: ICC, intra‐class correlation coefficient; PSIC, parasternal intercostal; SMU, single motor unit.

### 4.2 Intra‐oesophageal catheter

The unique anatomy of the diaphragm means that its crural portion can be recorded from its internal surface using a catheter that is passed via the nose and swallowed such that the recording electrodes sit adjacent to the crural diaphragm (see Table 2). The design of the catheters has evolved over time, but generally, a multi‐pair catheter (see Jolley et al., 2009) is used to minimize artefact associated with diaphragm movement with changes in lung volume when only bipolar recordings are made (Gandevia & McKenzie, 1986). The EMG recordings from the multi‐pair catheters consist of five bipolar recordings, each equivalent to bipolar surface EMG recordings. Thus, muscle activity is normalized to activity (typically during maximal volitional inspiratory manoeuvres (e.g. Jolley et al., 2009; Reilly et al., 2011), but activity has also been normalized to resting breathing (Nguyen, Amirjani et al., 2020) to estimate neural respiratory drive.

**TABLE 2:** The technical principles underlying the assessment of respiratory muscle activity using an intra‐oesophageal catheter.

Technical principles	Intra‐oesophageal catheter recordings
Details for proper use (calibration, validation, range, sensitivity, reproducibility, variability minimization)	Calibration. Position the electrode correctly to minimize artefacts if the diaphragm moves along the catheter length. If normalizing to maximal EMG, perform a variety of maximal volitional inspiratory manoeuvres (e.g. Jolley et al., 2009; Reilly et al., 2011). If maximal manoeuvres are not possible (e.g. in critically ill patients; Murphy et al., 2011), consider normalizing to the compound muscle action potential evoked by supramaximal stimulation of the phrenic nerves (Jolley et al., 2009). Perform maximal manoeuvres in different postures, lung volumes etc., as necessary. Validation. Record ECG as a separate signal using bipolar surface electrodes for use as a trigger during artefact removal and/or confirm that EMG, not ECG, is measured during analysis. Ideally, check every measurement by eye and/or remove ECG before measurement with post‐processing techniques (for a review, see Jonkman et al., 2024). Wavelet techniques (Hudson et al., 2024; Nguyen, Amirjani et al., 2020) are preferred over ‘gating’ methods. Range. Set appropriate amplification to capture all activity in maximal manoeuvres and take care if the diaphragm length is likely to change. Sensitivity. Good, because electrodes are adjacent to muscle (independent of body mass index). A sampling rate of 2 kHz and a high‐pass filter of >20 Hz are suggested to minimize ECG‐, movement‐ and oesophageal peristalsis‐related artefacts. Reproducibility. Excellent inter‐ and intra‐subject for repeated measures (ICC 0.94), good inter‐rater analysis (ICC 0.71) (Jolley et al., 2009). Variability minimization. Position electrode correctly and conduct range of maximal manoeuvres.
Tips for effective use	Apply local anaesthetic spray to the nasal passages and posterior pharyngeal wall prior to insertion to minimize gag reflex during insertion. For longer protocols, additional sprays can be delivered. The position of the head can help passage of the catheter through the airway. If seated, the neck is flexed and head extended. Advance catheter in synchrony with dry swallows or small swallows of water. Tape the electrode securely at the nose, once positioned correctly. Remove ECG before analysis or take caution when analysing. This can be achieved using various techniques (see ‘Validation’ above), such as fixed sample entropy (fSampEn), which is less influenced by cardiac artefacts than conventional root mean square (Lozano‐Garcia et al., 2018).
How to avoid errors	Use a multi‐pair electrode to avoid artefactual changes in EMG amplitude and always optimize electrode position. Check position if posture or lung volume changes. If normalizing to maximal manoeuvres, be aware that the ability to activate the respiratory muscles voluntarily is diminished in some clinical populations (e.g. Newell et al., 1989).
Appropriate uses of techniques, with examples	Appropriate in clinical populations with compromized lung function, such as chronic obstructive pulmonary disease (Domnik et al., 2022; Jolley et al., 2015) and cystic fibrosis (Reilly et al., 2011), in whom intramuscular recordings might be unsuitable during resting breathing. Recommended for exercise protocols (e.g. cardiopulmonary exercise tests) with large lung volumes and movement (James et al., 2022) and overnight studies (e.g. Domnik et al., 2022). The best estimate of neural respiratory drive from the crural diaphragm, using a method that is acceptable in >95% of patients (Jolley et al., 2009).
Inappropriate uses of techniques, with examples	Assumption that crural diaphragm EMG amplitude accurately reflects all inspiratory muscles and overall amplitude of respiratory neural drive (Hudson & Catcheside, 2022; Nguyen, Amirjani et al., 2020; Niro et al., 2021).

### 4.3 Surface recordings

For the more superficial respiratory muscles, bipolar EMG recordings can be made with surface electrodes stuck on the skin over the muscle of interest. To assess neural respiratory drive, surface recordings from the second parasternal intercostal muscle are used because their anatomy and correlation with crural diaphragm activity (recorded using intra‐oesophageal electrodes) make them a good candidate (Reilly et al., 2011). Surface recordings are also made from the scalene muscles, usually to test inspiratory muscle reflexes, and from the lateral chest wall overlying the costal diaphragm, although the latter is more susceptible to crosstalk from adjacent muscles (see Table 3). In addition to monitoring respiratory muscle activity during breathing at rest (see Table 3), surface recordings are used for evoked responses, but these applications are not discussed here.

**TABLE 3:** The technical principles underlying the assessment of respiratory muscle activity using surface electrodes.

Technical principles	Surface EMG recordings
Details for proper use (calibration, validation, range, sensitivity, reproducibility, variability minimization)	Calibration. Normalize to maximal activity or activity in resting breathing. Note that maximal activity for some muscles is during non‐respiratory manoeuvres (see Hudson et al., 2024). Where possible, reduce background tonic activity in muscles by using a supportive chair. Validation. Always confirm electrode position with voluntary manoeuvres, such as sniffs for inspiratory muscles. Range. Set appropriate amplification to capture all activity in maximal manoeuvres. Sensitivity. Less sensitive for some muscles and body forms, such as breast and/or pectoralis muscle tissue for lower PSIC interspaces (Hudson et al., 2024). Recordings from some muscles are more suitable for measures of peak inspiratory amplitude rather the onset of inspiratory activity if crosstalk from expiratory muscles is present; for example, lateral chest wall recordings of costal diaphragm are not suitable for onset measures owing to contamination by expiratory muscles, whereas surface recordings from the second PSIC (Hudson et al., 2024) and scalene (Hug et al., 2006) muscles are appropriate. A sampling rate of 2 kHz is suggested. Reproducibility. For participants with ‘normal’ body mass index, raw and normalized (to maximal) PSIC measures are repeatable between visits, but raw measures differ for recordings within a visit (e.g. Bland–Altman bias for raw values are 0.63 µV for within‐visit recordings versus −0.21 µV for between visits) (MacBean, Hughes et al., 2016). Normalized, but not raw, measures vary between postures (e.g. the Bland–Altman bias between raw and normalized measures is greatest for the supine vs. seated posture) (see Williams et al., 2019). Variability minimization. Place electrodes in reproducible (standard) positions (see Jonkman et al., 2024). Our preferred position for the costal diaphragm for resting breathing is ∼40 mm inter‐electrode distance along the seventh or eighth intercostal space at the anterior axillary line (e.g. Hudson et al., 2024; McBain et al., 2015). Sensitivity. Depends on body composition and the distance between skin and muscle. The ECG artefact is ∼3 times larger for surface than intramuscular recordings at the same recording site, which might influence analysis (Demoule et al., 2003). Validity. Good for scalene compared with intramuscular recordings (Hug et al., 2006) and for second PSIC compared with the diaphragm using an intra‐oesophageal catheter (Reilly et al., 2011).
Tips for effective use	Measure the root mean square or integrated EMG signals to quantify the combined effect of both motor unit recruitment and rate coding.
How to avoid errors	Be aware of crosstalk from adjacent inspiratory and expiratory muscles and try to minimize it, for example, unload expiration using a two‐way valve if inspiration is loaded. Measure inspiratory increases only in activity (i.e. that above tonic activity or level of activity during expiration).
Appropriate uses of techniques, with examples	Inspiratory reflexes, including from the costal diaphragm (over lateral chest wall), in people with paralysed abdominal and/or intercostal muscles attributable to spinal cord injury (e.g. McBain et al., 2015). Best estimate of neural respiratory drive with surface recordings from the second PSIC muscles. Especially appropriate for repeated monitoring (Suh et al., 2015) because more acceptable than the intra‐oesophageal catheter. A non‐invasive method to assess the pattern of inspiratory muscle activity across the PSIC muscles, but using the onset of activity only (not amplitude measures that require normalization owing to variable depths of each muscle from the skin surface that affect amplitude) (Hudson et al., 2024).
Inappropriate uses of techniques, with examples	Protocols with pectoralis activation owing to crosstalk; for example, voluntary movements of the arms (see Tagliabue et al., 2021 and accompanying commentary). Estimate of neural respiratory drive during cycling exercise without accounting for changes in the background (tonic) level of EMG (see Ramsook et al., 2017 and accompanying commentary).

Abbreviation: PSIC, parasternal intercostal.

**FIGURE 3:**
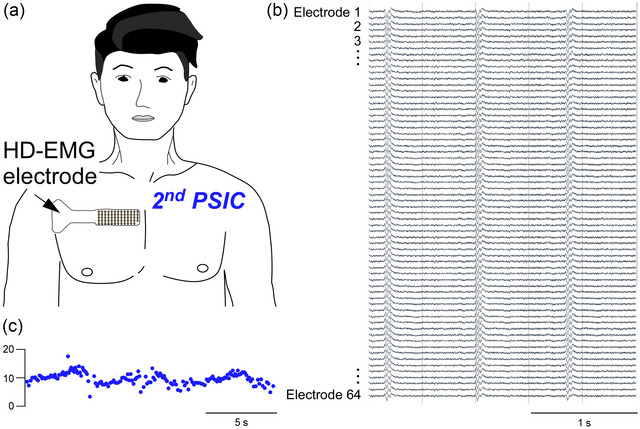
Pilot data of high‐density EMG recordings from the second parasternal intercostal muscle. (a) Example recordings from one healthy participant (male, aged 40 years) using a surface high density (HD)‐EMG grid (GR04MM1305, OT Bioelettronica, Italy) over the right second intercostal space close to the sternum. (b) Raw signals from 64 channels during resting breathing. The ECG is also evident on each channel. (c) The instantaneous discharge plot of a single motor unit discriminated from these recordings. It has a continuous discharge with inspiratory modulation (presumably, because breathing parameters were not recorded). Data were collected in Sydney, NSW, Australia.

Recent innovations of matrix or multi‐channel recordings [i.e., high‐density (HD)‐EMG] enable single motor unit recordings with a surface electrode in limb muscles. As a result, they ‘upset the balance’ (in a good way!) of the hierarchy of EMG methodologies that was introduced in the ‘Myths’ section, where the best estimate of neural respiratory drive (single motor units) must be balanced against the safety/practical considerations of the technique. These methods are increasingly used to record the activity of SMUs in limb muscles non‐invasively but are yet to be assessed thoroughly for use for respiratory muscles as a non‐invasive way to make precise recordings of motoneurone output (see Figure 3).

### 4.4 Non‐EMG methods

There are non‐EMG methods that use intrathoracic pressure measurements to infer respiratory muscle activity (see Hudson & Laveneziana, 2015) or ‘breathing effort’ (Gell et al., 2024), but pressures might not reflect respiratory muscle activity and neural respiratory drive, especially in the setting of severe respiratory mechanical impairment (Hudson & Laveneziana, 2015). The ‘breathing effort’ technique goes beyond simply reporting the pressures generated during breathing or breathing attempts (e.g. in the case of obstructive sleep apnoea) and converts pressure measures to attempted ventilation using first principles of respiratory mechanics (Gell et al., 2024). This reflects the overall output of all inspiratory muscles when balanced against the mechanical constraints of the system and can be compared against real ventilation measured by a pneumotachograph to expose the respiratory impairment (Hudson & Catcheside, 2022). How estimates of ventilation from this method compare with EMG assessments of neural respiratory drive are unknown. The change in mouth pressure after 0.1 s of an airway occlusion (P0.1) has been used for 50 years as a non‐invasive method to estimate the output from respiratory centres (i.e., neural respiratory drive), but again, it is only an estimate and is affected by factors such as mechanical impairments (American Thoracic Society/European Respiratory Society, 2002; Laveneziana et al., 2019).

Recently, surface mechanomyography (sMMG) recorded over the second parasternal intercostal space (sMMG_para_) and over lower intercostal spaces (sMMG_lic_), using skin‐surface triaxial accelerometers, has been used to measure inspiratory muscle fibre vibration during contraction and has been proposed to provide non‐invasive indices of inspiratory muscle force generation. When used in combination with surface respiratory muscle EMG, sMMG measures hold promise as non‐invasive indices of mechanical efficiency and neuromechanical coupling and have been evaluated in healthy subjects (Lozano‐Garcia et al., 2018) and as indices of disease severity in chronic obstructive pulmonary disease (Lozano‐Garcia et al., 2022).

## 5 CONCLUSIONS

**FIGURE 4:**
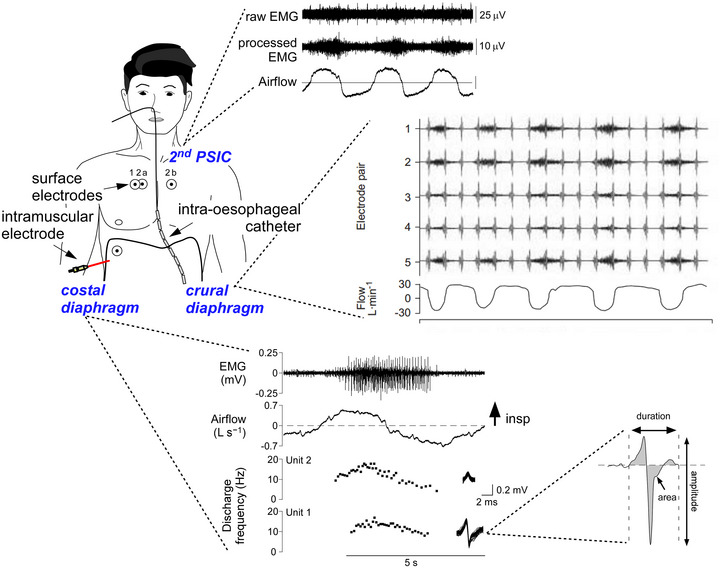
Estimating neural respiratory drive from recordings of respiratory muscle activity: the most appropriate muscles and methods. Example EMG data from three methodologies for recording inspiratory muscle activity as an estimate of neural respiratory drive. The second parasternal intercostal (PSIC) muscles are suitable for surface recordings of EMG owing to their superficial location (top inset). EMG in the crural diaphragm can be measured by intra‐oesophageal catheter (top, right inset). The best estimate of neural respiratory drive during resting breathing is determined by recordings of single motor units from the costal diaphragm (bottom inset), which can also be used to assess neurogenic changes (duration, area, amplitude of motor unit potentials; bottom, right inset). Along with stepwise increases in the accuracy of these measures to estimate neural respiratory drive comes increased safety and practical considerations, such as difficulty in participant recruitment. Figure adapted from: AL Hudson et al. (2024); Jolley et al. (2009); Nguyen et al. (2019).

It is crucial to assess respiratory muscle activity to understand respiratory physiology, pathophysiology of diseases and injuries, and for clinical investigations. We have made recommendations regarding which inspiratory muscles to record and which method to use (Figure 4) depending on the protocol, setting and participant population. These recordings must always be interpreted in the knowledge that they are an estimate of neural respiratory drive. Non‐EMG methods can also estimate neural respiratory drive. Emerging techniques (e.g. HD‐EMG) might provide a non‐invasive way to obtain the ‘best estimate’ of neural respiratory drive we have in humans (i.e., single motor unit output that represents the direct output of respiratory motoneurones), although, arguably, this is still an indirect measure of neural respiratory drive because the output measured will depend on the intrinsic properties of the motoneurones at any given time. In addition, motoneurone output will also depend on descending connectivity. For example, with muscle paralysis in spinal cord injury, neural respiratory drive could be large but unable to access the motoneurone pool.

Nonetheless, together with measures of respiratory muscle pressure generation, respiratory muscle EMG techniques facilitate greater understanding of the neurophysiology of breathlessness perception in health and in respiratory disease (James et al., 2022). Crural diaphragm EMG is increasingly recognized to provide an index of neural respiratory drive that is closely related to patient‐reported intensity of breathlessness (e.g. Jolley et al., 2015). Advances in assessment of respiratory muscle function have potential to be useful in the following clinical settings: monitoring diaphragm weakness as a prognostic factor in motor neuron disease (Polkey et al., 2017), monitoring acute exacerbations of chronic obstructive pulmonary disease (Suh et al., 2015), weaning from mechanical ventilation in critical care settings (Hadfield et al., 2020) and monitoring patients who cannot perform standard clinical lung function tests reliably (MacBean, Jolley et al., 2016). The applications will expand with further advancement of methods to measure respiratory muscle activity in humans.

## AUTHOR CONTRIBUTIONS

Anna L. Hudson: Conceptualization, investigation, supervision, visualization, writing—original draft preparation, writing—review and editing. Caroline J. Jolley: Investigation, writing—review and editing. Simon C. Gandevia: Conceptualization, investigation, writing—review and editing. Jane E. Butler: Conceptualization, investigation, writing—review and editing. All approved the final version of the manuscript and agree to be accountable for all aspects of the work in ensuring that questions related to the accuracy or integrity of any part of the work are appropriately investigated and resolved. All persons designated as authors qualify for authorship, and all those who qualify for authorship are listed.

## CONFLICT OF INTEREST

None declared.

## Data Availability

Data sharing is not applicable to this article because no datasets were generated or analysed during the present study.
